# EGFR-TKI治疗非小细胞肺癌中枢神经系统转移的进展

**DOI:** 10.3779/j.issn.1009-3419.2016.08.02

**Published:** 2016-08-20

**Authors:** 英华 金, 涛 信

**Affiliations:** 150001 哈尔滨，哈尔滨医科大学附属第二医院 The Second Afliated Hospital of Harbin Medical University, Harbin 150001, China

**Keywords:** 肺肿瘤, 表皮生长因子受体, EGFR-TKIs, 中枢神经系统转移, Lung neoplasms, Epidermal growth factor receptor, EGFR-tyrosine kinase inhibitors, Central nervous system metastases

## Abstract

约50%的非小细胞肺癌会出现中枢神经系统转移，从而导致不良预后。非小细胞肺癌患者中存在表皮生长因子受体（epidermal growth factor receptor, *EGFR*）突变，这部分患者对EGFR酪氨酸激酶抑制剂（EGFRtyrosine kinase inhibitors, EGFR-TKI）的治疗显示出了良好的耐受性及疗效。EGFR-TKI对非小细胞肺癌中枢神经系统转移也显示出了一定的疗效。本文针对EGFR-TKI药物对于非小细胞肺癌中枢神经系统转移的治疗进展进行综述。

## Abstract (en)

Approximately half of all patients with non-small cell lung cancer (NSCLC) develop central nervous system metastases during the course of their disease which indicate poor prognosis. A part of NSCLC patients demonstrates activating epidermal growth factor receptor gene (*EGFR*) mutations who represent effectiveness and well tolerance of EGFRspecific tyrosine kinase inhibitors (TKIs) therapy. Although the systemic efcacy of targeted agents is established, the efcacy of central nervous system (CNS) metastases is not as well characterized. In this article, we review recent data on the use of EGFR inhibitors for treatment of patients with NSCLC and CNS metastases.

肺癌目前依然是美国死亡率最高的肿瘤^[1]^。其中非小细胞肺癌(non-small cell lung cancer, NSCLC)占80%-85%, 且大多数发现时已不能切除或转移^[2, 3]^。新治疗方案的出现提高了患者生存期, 但对于转移的患者5年生存率依然很低^[4]^。

NSCLC中枢神经系统(central nervous system, CNS)转移的发生率高达30%-50%, 转移相关神经症状严重影响了患者的生活质量, 脑转移发生也意味着不良的预后, 其中未接受治疗的患者中位生存期仅1个月, 接受激素治疗的为2个月, 接受其他治疗为2个月-6个月^[5-13]^。

近年来表皮生长因子受体酪氨酸激酶抑制剂(epidermal growth factor receptor tyrosine kinase inhibitors, EGFR-TKIs)为NSCLC患者带来了新的治疗希望。NSCLC患者中的*EGFR*突变在亚裔人群发生率约30%-40%, 白种人约10%^[14-17]^。EGFR-TKIs治疗*EGFR*突变的NSCLC反应率高达56%-74%, 中位无进展生存期(progression-free survival, PFS)为10个月-14个月, 远高于化疗^[18-22]^。EGFR-TKIs药物在全身治疗获得了良好疗效, 但其对CNS转移疗效有限。本文针对新一代EGFR-TKIs药物对于NSCLC的CNS转移治疗进展进行综述。

## 1 NSCLC CNS转移的标准治疗

血脑屏障作为物理屏障的存在阻碍了化疗药物进入CNS, 有研究^[23-27]^显示NSCLC全身化疗对脑转移有效, 但其疗效不理想。

目前, 手术切除及放疗是临床上治疗脑转移最常用的方法。脑脊液相关的治疗(例如脑脊液化疗)被用于脑膜转移的患者, 但这部分患者中位生存期亦仅2个月-3个月^[28]^。

## 2 NSCLC CNS转移的EGFR-TKIs治疗及血脑屏障的相关作用

多项随机临床研究结果^[18, 22, 29]^证实存在基因突变的NSCLC患者一线EGFR-TKIs治疗反应率高达70%-80%, 中位PFS约12个月, 而且与化疗相比明显提高了生活质量。

目前EGFR-TKIs相关的研究很多, 但针对CNS转移的研究有限^[30]^。而CNS是接受EGFR-TKIs治疗患者的常见复发部位, 接受EGFR-TKIs治疗后局部病灶控制良好的NSCLC首次复发部位为CNS的患者比例达30%^[31-34]^。

回顾性研究结果^[35]^显示一线接受EGFR-TKIs治疗与一线接受化疗的患者相比, 12个月内发生CNS进展的患者比例分别为6%与19%。*EGFR*突变的脑转移患者与野生型相比表现出更好的预后, 这可能与*EGFR*突变患者对颅内放疗更敏感或与颅内EGFR-TKIs的活性有关^[36, 37]^。一项关于厄洛替尼的回顾性研究显示*EGFR*突变与*EGFR*野生型或未检测的患者相比, 脑转移病灶进展的时间分别为11.7个月与5.8个月, 对照组85%的患者接受了放疗, 而*EGFR*突变的患者中仅有16%接受了放疗^[38]^。吴一龙等^[39]^进行了一项前瞻性研究, 该研究给予伴有无症状性脑转移的亚洲NSCLC二线厄洛替尼治疗, 研究组16.7%的患者为*EGFR*突变, 31.3%野生型, 52%未知突变状态, 结果显示全部患者颅内病灶中位PFS为10.1个月, 总的PFS为9.7个月, 其中*EGFR*突变患者中位PFS为15.2个月, *EGFR*野生型4.4个月。

EGFR-TKIs对于脑转移灶的治疗有效可能与脑脊液中检测到EGFR-TKIs浓度相关, 脑脊液中药物进入颅内转移病灶可能是其大幅改善存在基因改变的NSCLC患者预后的原因。但EGFR-TKIs对CNS转移的疗效却远不及外周病灶疗效, 这可能与血脑屏障限制了外周EGFR-TKIs进入脑脊液相关^[40]^。

血脑屏障结构中存在的内皮细胞间紧密连接降低了脑循环系统的药物渗透率, 因此限制药物有效进入CNS及颅内病灶^[41, 42]^。血脑屏障中内皮细胞还存在着多种转运蛋白, 其中药物外排转运体蛋白为中枢代谢物及神经毒物的清除系统, 因此可以被其识别的药物很难进入CNS, 例如P糖蛋白(P-glycoprotein, P-gp)及乳腺癌耐药蛋白可以阻碍厄洛替尼通过血脑屏障进而减弱厄洛替尼治疗颅内转移灶的疗效^[43-45]^。

研究显示在肿瘤生长及转移的过程中会对血脑屏障产生一定程度的损伤, 但仍不足以增加包括单克隆抗体及EGFR-TKIs在内的大分子药物的通过。Weber等^[46]^研究了1例NSCLC伴随多发脑转移的患者, 该研究使用碳11标记的厄洛替尼治疗, 然后进行正电子发射计算机体层成像(positron emission tomography-computed tomography, PET-CT), 结果显示厄洛替尼可进入脑转移病灶。虽然EGFR-TKIs可以通过血脑屏障进入CNS, 但其脑脊液中药物浓度仍远远低于外周循环浓度。Togashi等^[47]^研究显示厄洛替尼及其活性代谢物OSI-420在NSCLC脑转移患者脑脊液中的浓度分别为血浆浓度的5.1%与5.8%, 且脑脊液中的EGFR-TKIs浓度与血浆中药物浓度呈线性关系。Jackman等^[48]^报道了1例通过提高靶向治疗药物剂量使脑脊液药物浓度升高并使脑转移病灶减小的病例。Jackman等^[49]^进一步研究了10例接受厄洛替尼脉冲式大剂量治疗的病例, 结果显示CNS反应率为10%, 中位CNS的PFS为1.7个月, 该研究结果显示加大剂量可以增加疗效, 但如何平衡大剂量药物产生的副作用及治疗疗效之间的关系仍需进一步研究。

## 3 二代EGFR-TKIs

二代EGFR-TKIs阿法替尼及达可替尼为EGFR的不可逆抑制剂, 其中阿法替尼已经获批用于晚期肺癌一线治疗^[50]^。Petra Hoffknecht等^[51]^研究显示接受阿法替尼治疗CNS转移的31例NSCLC患者中42%(13例)评价为部分缓解(partial response, PR), 39%(12例)评价为病情稳定(stable disease, SD), 仅有19%(6例)为疾病进展(progressive disease, PD)。

达可替尼的二期临床试验显示一线接受治疗的45例*EGFR*突变NSCLC患者总反应率为76%, 中位PFS为18.2个月, 另一项三期临床试验比较达可替尼与吉非替尼一线治疗*EGFR*突变的NSCLC, 但是该研究并未入组脑转移或脑膜转移的患者^[52]^。正在进行的NCT02047747为一项评估达可替尼治疗脑转移肿瘤的二期临床试验, 虽然此试验并非针对*EGFR*突变的NSCLC, 但是可协助观察该药物的脑脊液药代动力学情况^[53]^。

## 4 三代EGFR-TKIs

三代药物用于EGFR-TKIs治疗后出现获得性耐药突变(如T790突变)的患者。包括AZD9291, rociletinib(CO-1686), HM61713等, 其中AZD9291及CO-1686治疗存在T790突变的患者反应率分别为61%和64%^[54, 55]^。

Sequist等^[55]^研究显示1例CNS转移的患者接受CO-1686治疗后有效。Kim等^[56]^进行的临床前试验显示AZD-9291在一部分脑转移患者中有效, 但关于具体的CNS治疗效果的反应率现在尚无临床试验报道。HM61713对T790突变的患者具有一定的疗效并且显示了良好的安全性, 但其目前的临床试验尚在进行中, 尚无关于CNS活性的相关报道^[57]^。

## 5 AZD3759

AZD3759是一种针对CNS转移而设计的口服EGFR-TKIs, 研究者通过调整药物化学分子的相关性质使药物更利于穿过血脑屏障。在猴脑进行的药物追踪试验显示该药物可以有效进入猴的脑脊液, 脑转移动物模型实验进一步证实AZD3759对脑转移灶疗效明显。此药物目前正在进行临床一期试验^[58]^。

## 6 展望

现今分子靶向药物治疗发展迅速, 从最初的EGFR, 到后来发现间变性淋巴瘤激酶(anaplastic lymphoma kinase, ALK), 其他包括*ROS1*及*RET*基因异常及*HER2*和*BRAF*基因等都已经成为了临床上重要的分子靶点^[59]^。Preusser等^[60]^进行了一项针对肺腺癌脑转移基因突变谱的研究, 在该研究检测的48个肿瘤相关基因中, 其中29个(60.4%)至少在一个脑转移样本中被检出, 检测的76例脑转移样本中有64例(84.2%)至少存在一种基因突变, 其中最常见的为*TP53*(46.1%), *KRAS*(38.2%), *CDKN2A*(22.4%), 其他治疗药物相关的基因突变包括*EGFR*(3.9%), *PIK3CA*(2.6%), *BRAF*(1.3%), *SMO*(1.3%)。

目前仍在进行的临床试验包括埃克替尼及其他EGFR-TKIs联合或不联合放疗治疗脑转移的治疗, 但尚无肺癌脑转移突变基因相关的靶向药物治疗研究, 针对肺癌脑转移突变基因的个体化靶向药物治疗的研究更是空白, 期待未来能有更多更深入的研究进一步开展, 使未来肺癌脑转移真正实现基因个体化治疗。
